# Impact of HCMV Infection on NK Cell Development and Function after HSCT

**DOI:** 10.3389/fimmu.2013.00458

**Published:** 2013-12-13

**Authors:** Mariella Della Chiesa, Michela Falco, Letizia Muccio, Alice Bertaina, Franco Locatelli, Alessandro Moretta

**Affiliations:** ^1^DI.ME.S. Dipartimento di Medicina Sperimentale, Centro di Eccellenza per la Ricerca Biomedica, Università di Genova, Genova, Italy; ^2^Istituto Giannina Gaslini, Genova, Italy; ^3^Dipartimento di Onco-Ematologia Pediatrica, Ospedale Bambino Gesù, Roma, Italy; ^4^University of Pavia, Pavia, Italy

**Keywords:** human NK cells, HCMV infection, NKG2C, KIR, hematopoietic stem cell transplantation

## Abstract

Natural Killer (NK) cell function is regulated by an array of inhibitory and activating surface receptors that during NK cell differentiation, at variance with T and B cells, do not require genetic rearrangement. Importantly, NK cells are the first lymphocyte population recovering after hematopoietic stem cell transplantation (HSCT). Thus, their role in early immunity after HSCT is considered crucial, as they can importantly contribute to protect the host from tumor recurrence and viral infections before T-cell immunity is fully recovered. In order to acquire effector functions and regulatory receptors, NK cell precursors undergo a maturation process that can be analyzed during immune reconstitution after HSCT. In this context, the occurrence of human cytomegalovirus (HCMV) infection/reactivation was shown to accelerate NK cell maturation by promoting the differentiation of high frequencies of NK cells characterized by a KIR^+^NKG2A^−^ and NKG2C^+^ mature phenotype. Thus, it appears that the development of NK cells and the distribution of NK cell receptors can be deeply influenced by HCMV infection. Moreover, in HCMV-infected subjects the emergence of so called “memory-like” or “long-lived” NK cells has been documented. These cells could play an important role in protecting from infections and maybe from relapse in patients transplanted for leukemia. All the aspects regarding the influence of HCMV infection on NK cell development will be discussed.

## Introduction

Natural killer (NK) cells represent crucial effectors in innate immune response to viral infections and tumors. NK cell function is regulated by an array of germline-encoded surface receptors, that, upon interaction with their ligands, transmit either inhibitory or activating signals (1–3).

Most human NK cells express inhibitory receptors specific for HLA-class I molecules, including the Killer Ig-like Receptors (KIRs), able to distinguish among different HLA-A, -B, and -C allotypes (4), and the CD94/NKG2A heterodimer, specific for HLA-E (5). These receptors allow NK cells to spare HLA-class I^+^ autologous normal cells and to kill cells in which HLA class I expression is down-regulated (e.g., by tumor transformation or viral infection) or cells expressing non-self HLA class I alleles unable to engage inhibitory KIRs (e.g., in the allogeneic transplant settings).

Activating counterparts of the inhibitory receptors specific for HLA class I molecules can be expressed by NK cells. In particular, activating KIRs, including KIR2DS1, KIR2DS2, and KIR3DS1, are highly homologous to their inhibitory counterparts in the extracellular domain, but are characterized by a short cytoplasmic tail lacking Immunoreceptor Tyrosine-Based Inhibitory Motifs (ITIM). On the other hand, activating KIRs interact with DAP-12, an adaptor signaling molecule carrying an Immunoreceptor Tyrosine-Based Activating Motif (ITAM) that can induce NK cell activation (6). So far, the HLA class I specificity of activating KIRs has been clearly demonstrated only for KIR2DS1 and KIR2DS4 (6–8). KIR genes are located on chromosome 19 and are inherited as haplotypes. Two basic KIR haplotypes can be found in the human genome: group A haplotypes, which have a fixed number of genes that encode inhibitory receptors (with the exception of the activating receptor KIR2DS4) and group B haplotypes, which have variable gene content, including additional activating KIR genes (4, 9).

Another HLA class I-specific activating receptor is represented by the HLA-E-specific CD94/NKG2C heterodimer. At variance with its inhibitory counterpart CD94/NKG2A, which contains an ITIM in the NKG2A cytoplasmic domain, NKG2C, like the activating KIRs, lacks ITIM and is associated with DAP-12 (9).

Human NK cells mainly differentiate in the bone marrow (BM) from CD34^+^ hematopoietic stem cells (HSCs) through discrete stages of development. However, recent studies suggest that different sites, such as secondary lymphoid compartments (SLCs) (10), human decidua (11), thymus (12), or fetal and adult liver (13) can be involved in this process. Two main subsets of mature NK cells with distinct functional and phenotypic properties have been described: the CD56^bright^ CD16^−/low^ and the CD56^dim^ CD16^+^ subsets. CD56^bright^ NK cells typically express high levels of the receptor CD94/NKG2A, but low levels of KIR molecules. They are not abundant in peripheral blood, while dominate in SLCs for which they express specific homing receptors. CD56^bright^ NK cells produce high levels of immunoregulatory cytokines, but are poorly cytotoxic. In contrast, the CD56^dim^ subset is characterized by high surface expression of KIR receptors, is largely represented in peripheral blood and is highly cytotoxic against tumor and virus-infected targets (14). A recent study shows that CD56^dim^ NK cells may also release high amounts of IFN-γ very early after activation (15). In addition, a third subset, characterized by a CD56^−^CD16^+^ surface phenotype, exists, but is rare in healthy individuals and represents a small percentage of total NK cells. However, expansion of CD56^−^ NK cells have been described in HIV and HCV chronically infected subjects (16, 17) and also in recipients of hematopoietic stem cell transplantation (HSCT) (18, 19).

The developmental relationship between CD56^bright^ and CD56^dim^ has been long debated; however, recent reports suggest that CD56^dim^ derive from CD56^bright^ cells. Thus, it has been shown that in CD56^bright^CD16^−^ cells telomeres are significantly longer than in CD56^dim^CD16^bright^ cells (20). In addition, when CD56^bright^ NK cells were infused in NOD/SCID mice, they progressed toward the CD56^dim^ phenotype (21). In line with these observations the first wave of NK cells that reconstitute after HSCT is represented by the CD56^bright^ NK cell subset. Only later on CD56^dim^ NK cells appear in the blood and are initially characterized by high CD94/NKG2A expression. The complete maturation of CD56^dim^ NK cells involve the progressive loss of CD94/NKG2A and the acquisition of combinations of KIR molecules, in order to form a relatively stable repertoire which is mainly genetically determined, but influenced by the HLA class I genotype (4, 22–25). During their late stages of differentiation, NKG2A^−^KIR^+^ CD56^dim^ NK cells progressively acquire a CD57^+^ CD94^low^ CD62L^neg^ phenotype (26–29). In order to become fully competent NK cells have been shown to require recognition of self HLA class I molecules during maturation, a phenomenon referred to as “licensing” or “education” (30–32). However, unlicensed NK cells lacking inhibitory receptors specific for self HLA molecules (either KIR or NKG2A/CD94) do exist, but are hyporesponsive (31, 33). Thus, in a self environment the NK cell receptor repertoire will ensure self tolerance as each functional NK cell expresses at least one inhibitory receptor specific for self HLA-I molecules. In contrast, in allogeneic settings, NK cells may kill allogeneic cells expressing HLA-I non-self molecules. The existence of “alloreactive” NK cells can be particularly important in HSCT (34). Since HSCT is a widely employed treatment used to cure malignant disorders such as acute leukemia, these alloreactive NK cells can greatly contribute to the eradication of residual tumor cells (Graft vs. Leukemia effect), prevent Graft vs. Host Disease (GvHD), and improve engraftment by the killing of recipient dendritic cells (DC) and T-cells, respectively (35, 36).

## NK Cell Responses to HCMV Infection

Natural killer cells can importantly contribute to immune responses against viral infections. Indeed, in patients with NK cell deficiency a higher susceptibility to herpesvirus infections has been observed (37–39).

Human cytomegalovirus (HCMV) is a β-herpesvirus that establishes a lifelong persistent infection (40). In immunocompetent hosts, HCMV infection is usually asymptomatic, but reactivation becomes an important cause of morbidity in primary or acquired immunodeficiencies and immunosuppressed patients particularly in transplant recipients. In healthy individuals both T-cell and NK cells are involved in controlling HCMV infection (41).

During the host-HCMV interplay, NK cells are likely to receive stimuli from infected cells or other immune cells that can modulate their phenotype and function. In particular it has been shown that HCMV is capable of shaping the NK cell receptor repertoire inducing the expansion of an NK cell subset expressing the activating NKG2C receptor. Remarkably, this expanded NKG2C^+^ NK cell subset found in HCMV seropositive individuals is also characterized by a mature phenotype, mostly KIR^+^NKG2A^−^ (42). This finding was observed both in healthy individuals and in patients with different pathological conditions. Indeed, increased proportions of KIR^+^NKG2A^−^NKG2C^+^ NK cells have been described in subjects who experienced different viral infections, including HIV (43, 44), Chikungunya virus (45), Hantavirus (46), HBV and HCV (47). However, it is conceivable that HCMV infection/reactivation, which may occur in chronically infected subjects, may be responsible for the induction of such NK cell phenotype in these patients. NKG2C^+^KIR^+^ NK cell expansions have been described also in congenital immunodeficiencies where this NK cell subset has been proposed to play a relevant role in the resolution of HCMV infection (48). Together, these data suggest that the NKG2C receptor could play a crucial role in HCMV recognition and in promoting the expansion and/or maturation of NKG2C^+^ cells, as well as in the control of infection.

The mechanism responsible for the NK cell expansion described is still unclear. It has been suggested that NK cells could be stimulated by HCMV-infected targets, through the heterodimer CD94/NKG2C. In this context, HCMV-infected fibroblasts have been shown to favor the expansion of NKG2C^+^ NK cells, cultured in the presence of either IL-15 or IL-2. MAb-mediated masking of CD94 could inhibit this selective expansion (49). Other studies reported that NKG2C^+^ NK cells can undergo proliferation when co-cultured with HLA-E-transfected 721.221 lymphoblastoid cells (50, 51) or K562 cell lines (46). It is possible that HCMV-infected targets may express ligands interacting with NKG2C^+^ NK cells, thus inducing their activation and proliferation. Remarkably, the signal peptide of the HCMV UL40 protein stabilizes HLA-E expression on HCMV-infected fibroblasts, while other HCMV-derived peptides (including US2, US3, US6, US10, and US11) dampen the surface expression of classical HLA class I molecules (52). These observations suggest that, during the interaction with HCMV-infected cells, the expansion of mature NK cells expressing inhibitory self KIRs could be favored because of the lack of inhibitory interactions with classical HLA class I molecules. Moreover, the stabilization of HLA-E, while favoring the expansion of NKG2C^+^ cells, would inhibit that of cells expressing NKG2A. This is in line with a recent study showing that a past HCMV infection in healthy individuals is strongly correlated with expansion of NKG2C^+^ NK cells expressing inhibitory self KIRs (i.e., educated NK cells expressing KIRs specific for self HLA class I molecules) (51).

However, direct evidence for the specificity of NKG2C for HLA-E molecules loaded with viral peptides, or for an unknown ligand of either host or viral origin expressed by HCMV-infected cells is missing or limited. It would be important to analyze the peptides that *in vivo* are bound to HLA-E in HCMV-infected cells and to verify their role in inducing NKG2C-mediated NK cell recognition. In this context, by the analysis of HCMV UL40 sequences isolated from HSCT recipients undergoing HCMV reactivation, it has been shown that UL40 is characterized by a certain degree of polymorphism that could modulate NK cell-mediated recognition of HCMV-infected targets. In particular, some UL40 peptides derived from the signal sequence (i.e., HLA-E-binding peptides) encoded by HCMV isolates, are capable of both inhibiting NK cell lysis by NKG2A engagement and inducing NK cell activation through NKG2C triggering, whereas other forms of UL40 peptides do not stimulate NKG2C^+^ cells, but are still capable of inducing inhibitory responses via NKG2A (53). Whether such UL40 polymorphisms can affect the expansion of NK cells expressing NKG2C and/or virus clearance in HCMV-infected HSCT recipients is unknown.

Human NK cell responses to HCMV can also be elicited through direct recognition of HCMV virions by NK cells (54). After exposure to HCMV, NK cells become activated and produce IFN-γ. This anti-HCMV response involves the engagement of TLR2 on NK cells by viral particles and the endogenous release of IFN-β. However, direct recognition of HCMV is not sufficient to induce stable changes in NK cell receptor repertoire.

## HCMV Drives NK Cell Maturation Toward Highly Differentiated Stages in HSCT Recipients

The imprinting on NK cell phenotype induced by HCMV infection results particularly dramatic when T-cell immunity is impaired in the infected host, such as in chronically infected HIV patients (43, 44), congenitally immunodeficient individuals (48, 55) and patients undergoing HSCT.

In this context, two recent studies have shown that HCMV reactivation can promote a rapid NK cell development after umbilical cord blood transplantation (UCBT) (56, 57). NK cells achieved a full maturation more rapidly in HCMV-reactivating patients as compared to non-infected ones. In particular, NK cells isolated from HCMV-reactivating patients show low percentages of CD56^bright^ NK cell and high proportions of mature CD56^dim^ NK cells expressing the NKG2C^+^ NKG2A^−^ KIR^+^ Siglec-7^−^ CD57^+^ signature, at variance with non-reactivating patients that display a more immature phenotype (57). NKG2C^+^ CD56^dim^ NK cells were characterized by the expression of self-KIR and displayed full competence in terms of cytolytic activity and cytokine production. The frequency of mature KIR^+^NKG2C^+^ NK cells persisted and continued to increase after 1 year from HSCT in recipients who reactivated HCMV. This expanded and long-living NKG2C^+^ NK cell subset is clearly reminiscent of a population of Ly49H^+^ NK cells which expands in murine CMV (MCVM) infected mice and is responsible for disease clearance through the induction of a “memory-like” NK cell response. Thus, NKG2C^+^ NK cells, expanding after HCMV infection, could represent the human counterpart of murine “memory-like” NK cells. However, a clear recall response against HCMV-infected targets, mediated by human NKG2C^+^ NK cells, has not been shown yet.

Importantly, in mouse, the Ly49H receptor has been demonstrated to bind to the MCMV protein m157 which is expressed by infected cells (58).

**Figure 1:**
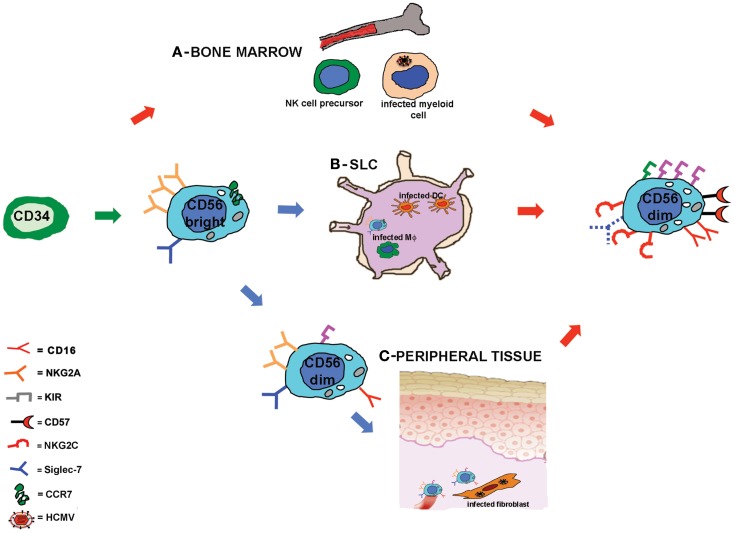
**Human cytomegalovirus accelerates NK cell maturation after HSCT: possible sites of NK cell-HCMV interplay**. Schematic representation of NK cell maturation from CD34^+^ HSC in recipients reactivating HCMV. NK cell development is rapidly driven toward a mature stage of differentiation characterized by a KIR^+^NKG2A^−^NKG2C^+^CD57^+^ Siglec-7^−^ surface phenotype. The signals driving NK cell maturation might be provided at different developmental stages in different sites: in **(A)** it is hypothesized that during NK cell differentiation in the bone marrow, NK cell precursors could interact with HCMV-infected myeloid cells; in **(B)** CD56^bright^ NK cells that express CCR7 can reach the secondary lymphoid compartment (SLC) where they could interact with HCMV-infected stromal cells, dendritic cells, or macrophages (Mϕ); in **(C)** circulating CD56^dim^ NKG2A^+^ NK cells could migrate to peripheral tissues and interact with HCMV-infected fibroblasts or endothelial cells.

The mechanisms lying behind this accelerated maturation and expansion of KIR^+^NKG2C^+^ NK cells after HSCT are not completely understood. As already discussed, developing NK cells could be stimulated by HCMV-infected targets, possibly through the heterodimer CD94/NKG2C. HCMV infection could exert its influence on NK cell development at different sites and/or stages of maturation (Figure 1). First, NK cell precursors could be stimulated by HCMV while differentiating in the BM where they may interact with cells of the myeloid lineage that can be infected by HCMV and that represent a reservoir of latent virus (41). Second, CD56^bright^ NK cells, that are characterized by the expression of the lymph node homing chemokine receptor CCR7 (59), can reach SLC (e.g., lymph node) and interact with infected antigen-presenting cells, DC, or macrophages, that would promote NK cell differentiation toward more mature CD56^dim^ stages. A third possibility is that circulating CD56^dim^ NK cells could migrate to peripheral tissues (59) (e.g., mucosal tissues) where HCMV infection can affect different cell types (fibroblasts, endothelial cells, epithelial cells) (41). In this microenvironment, NK cells would receive, directly or indirectly, proliferating and maturing signals from infected targets that would drive NK cells toward highly differentiated stages of maturation, characterized by the KIR^+^ NKG2C^+^ NKG2A^−^ signature. This last scenario better corresponds to *in vitro* data showing that CD56^dim^ NK cells proliferate in response to HCMV-infected fibroblasts (49). However, it is not clear so far whether this expanded NKG2C^+^KIR^+^NKG2A^−^Siglec-7^−^NK cell subset, developed in response to HCMV-derived stimuli, represents a long-living cell subset or is rather continuously replenished by novel mature NK cells.

Remarkably, in HCMV-reactivating patients a unusual CD56^−^CD16^+^Siglec-7^−^NK cell subset was detected (57) that was reminiscent of that described in viremic HIV-infected patients undergoing HCMV reactivation (60). The similarity between CD56^−^NK cells found in HCMV-infected UCBT recipients and those present in HIV-infected patients was also confirmed by a marked impairment in functional capabilities. This may be consequent, at least in part, to the low expression on CD56^−^ cells of several activating receptors such as NCRs and NKG2D, as also reported in chronically HIV-infected patients. It is conceivable that these hyporesponsive CD56^−^ NK cells are generated after HCMV infection when T-cell immunity is impaired. The accumulation of these phenotypically aberrant and hypofunctional NK cells could also support the concept that HCMV has a role in immunosenescence (61). However, it should be noted that, in HSCT recipients, the hypofunctional state of CD56^−^ NK cells could be reversed by exposure to exogenous cytokines, such as IL-2. This observation would suggest that the generation of these cells can be consequent to chronic stimulation together with lack of appropriate T-cell responses and cytokine production rather than to an irreversible process of aging induced by HCMV.

A distinguishing feature of both CD56^dim^ and CD56^−^ NK cells observed in HCMV-infected recipients is the dramatic down-regulation of Siglec-7. Although the mechanism/significance of this event is unknown, it represents, together with NKG2C up-regulation, the most typical marker of NK cell expansions promoted by HCMV infection. Whether Siglec-7 down-regulation could be a marker of “memory-like” NK cells has still to be investigated.

Human cytomegalovirus-induced NK cell populations with a “memory-like” surface phenotype may contribute not only to the control of virus infection, but also to the protection from leukemia relapses after HSCT. In this context, a recent study reported a correlation between early HCMV reactivation and reduction of leukemia relapse after allogeneic HSCT in adult patients (62). The HCMV-induced rapid maturation of functional NK cells could favor an NK cell-mediated anti-leukemic activity, especially in the case of a KIR-mismatched haplo-HSCT in which the fast differentiation of mature KIR^+^NKG2A^−^ NK cells could promote the emergence of anti-leukemic alloreactive NK cells.

“Memory-like” NKG2C^+^ NK cells have been also demonstrated to be transplantable employing unmanipulated adult grafts from HCMV seropositive donors (63). These donor-derived NK cells, contained in the graft, expanded not only in recipients undergoing HCMV reactivation, but also in seropositive recipients in the absence of detectable viremia. These NKG2C^+^ NK cells were capable of producing higher levels of IFN-γ as compared to NKG2C^+^ NK cells infused in seronegative recipients, suggesting that a subsequent exposure to viral antigens in the recipient can increase cytokine production, by inducing a “memory-like” response that might contribute to control of HCMV reactivation in these HCMV^+^ recipients. Of note, a recent paper suggested that NK cells exposed to multiple cytokines (a circumstance which likely occurs during an anti-viral immune response) can acquire a “memory-like” phenotype and would release higher amounts of IFN-γ following restimulation with cytokines or target cells (64).

NKG2C thus seems to play a central role in HCMV-induced responses by NK cells; however, recent reports indicated that also other NK receptors may be involved and that NKG2C could be dispensable. For example, in a cohort of children with or without congenital HCMV infection the deletion of one or two copies of NKG2C did not correlate with a higher incidence of HCMV infection (65). Further supporting the concept that NK receptors other than NKG2C can contribute to shape NK cell receptor repertoire, following HCMV infection, is a study reporting the expansion of NKG2A^−^NKG2C^−^ NK subsets expressing activating KIRs in a cohort of HCMV seropositive healthy individuals (51). In this context, a number of studies suggested that the presence of activating KIRs is protective against viral infections (66–69).

## Concluding Remarks

The development of NK cells reconstituting after transplantation can be profoundly affected by HCMV infection/reactivation, which is a common event in immunocompromised HSCT recipients. Although HCMV infection is cause of morbidity and mortality in such patients, it can also promote NK cell differentiation by accelerating the acquisition of a fully mature KIR^+^ NKG2A^−^ phenotype. Notably, in the case of KIR-mismatched haplo-HSCT, this phenomenon may be of particular benefit, since it results in rapid expansion of alloreactive NK cells (characterized in all instances by the KIR^+^NKG2A^−^ phenotype). These mature NK cells can display not only anti-leukemia activity but also important anti-viral activity that could be beneficial to HSCT recipients. Finally, the strong imprinting induced by HCMV on developing NK cells could be (cautiously) harnessed to design new adoptive NK cell based therapies.

## Conflict of Interest Statement

Alessandro Moretta is a founder and shareholder of Innate-Pharma (Marseille, France). The remaining authors declare no conflicts of interest.
